# Bacterial biofilm composition in healthy subjects with and without caries experience.

**DOI:** 10.1080/20002297.2019.1633194

**Published:** 2019-06-26

**Authors:** Kyrill Schoilew, Helena Ueffing, Alexander Dalpke, Björn Wolff, Cornelia Frese, Diana Wolff, Sébastien Boutin

**Affiliations:** a Department of Conservative Dentistry, School of Dental Medicine, University Hospital Heidelberg, Heidelberg, Germany; b Department of Infectious Diseases, Medical Microbiology and Hygiene, University Hospital Heidelberg, Heidelberg, Germany; c Institute of Medical Microbiology and Hygiene, Technical University Dresden, Dresden, Germany; d Department of Conservative Dentistry and Periodontology, Center of Dentistry, Oral Medicine and Maxillofacial Surgery, University Hospital Tübingen, Tübingen, Germany; e Translational Lung Research Center Heidelberg (TLRC), German Center for Lung Research (DZL), University of Heidelberg, Heidelberg, Germany

**Keywords:** Dental caries, dental plaque, dental health, oral microbiome, 16S rRNA

## Abstract

**Objective:**The composition of the oral microbiome differs distinctively between subjects with and without active caries. Still, caries research has mainly been focused on states of disease; aspects about how biofilm composition and structure maintain oral health still remain widely unclear. Therefore, the aim of the study was to compare the healthy oral microbiome of caries-free adult subjects with and without former caries experience using next generation sequencing methods.

**Methods:** 46 samples were collected from subjects without any signs of untreated active caries. Samples of pooled supragingival plaque from 19 subjects without caries experience (NH; DMFT = 0) and 27 subjects with ‘caries experience’ (***CE;*** DMFT > 0 [F(T)> 0; D(T)= 0]) were analyzed by 16S ribosomal RNA amplicon sequencing.

**Results:** Subjects with caries experience did not exhibit a dramatically modified supragingival plaque microbiome. However, we observed a slight and significant modification between the two groups, validated by PERMANOVA (***NH*** vs. ***CE:*** R2 0.04; p= 0.039). The composition of the microbiome of subjects with caries experience indicates a tendency to lower α-diversity and richness. Subjects without caries experience showed a significant higher evenness compared to patients with previous caries. LDA effect size (LEfSe) analysis demonstrated that the genus *Haemophilus* is significantly more frequent in patients with caries experience. For the group without caries experience LefSe analysis showed a set of 11 genera being significantly more frequent, including *Corynebacterium, Fusobacterium, Capnocytophaga, Porphyromonas, Prevotella,*and *Leptotrichia.*

**Conclusion:** The analysis of the oral microbiome of subjects with and without caries experience indicates specific differences. With the presence of *Corynebacterium* and *Fusobacterium* subjects without caries experience exhibited more frequently organisms that are considered to be main actors in structural plaque formation and integration. The abundance of *Corynebacterium* might be interpreted as a signature for dental health.

## Introduction

Recent technological and methodological advances provide a growing insight into the high complexity of the oral microbiome [1,2]. The oral cavity is a heterogeneous environment comprising variable habitats for microbial colonization [3], hosting the second most diverse bacterial population in the human body [4]: So far more than 700 bacterial species have been detected colonizing dental hard tissues and the oral mucosa with high intra- and interindividual variability [5].

In the last decades, caries research underwent several paradigm shifts from specific [6–9] and non-specific [10,11] plaque hypotheses to the currently prevailing ecological plaque hypothesis [12]. Accordingly, oral health is considered a finely tuned homeostasis between host and oral microorganisms. Furthermore, microbial homeostasis of the oral cavity is also providing important benefits to the host on a systemic level [13]. A disruption of the microbial homeostasis as a result of complex interplay between bacterial species, host and ecological factors can lead to virulent conditions – being coined by manifestation of specific pathogenic bacteria and resulting in oral diseases such as caries, gingivitis and periodontitis.

Initially, *Streptococcus mutans* was considered the key pathogen within the caries process [6]. By now, the association between caries and the presence of *S. mutans* has been confirmed. However, it is known that caries can develop in the absence of *S. mutans* as well [14–17]. Molecular and culture-independent approaches revealed that other acid-tolerating and acid-producing bacteria like *Lactobacillus, Actinomyces, Bifidobacterium, Veillonella, Propionibacterium* and *Atopobia* can complement or substitute *S. mutans* within the caries process [15,16,18,19]. Also, temporal changes in the microbiota associated with caries might be an additional variable. Up to now, research in the area of the oral microbiome has mainly focused on states of disease, revealing a tendency to characterize oral health in general as the mere absence of pathogenic species [20]. Interactions between host- and microbe-derived factors maintaining homeostasis are widely unclear [2,20]. Potentially cariogenic bacteria might also be found at healthy sites in low levels, which are clinically irrelevant and therefore not detrimental to microbial homeostasis, and thus impeding a clear separation between the state of health and disease [13].

In the past years, improved next generation sequencing techniques generated an increasing insight, especially into communities’ shift and involved key pathogens connected with progression towards disease. As a result, some long-held caries paradigms have been revised. A state associated with caries is characterized by a loss of community balance and diversity, leading to the predominance of few cariogenic species [1,2]. Nevertheless, essential aspects about how biofilm composition and structure maintain oral health are still unclear [21]. As a consequence, considerable limitations remain in the assessment of the individual caries risk. Despite more than a century of caries research, the caries experience of the individual is still the best single predictor for future caries development [22–25]. Therefore, adults without caries experience appear to have the lowest caries risk. In contrast, comparatively healthy subjects with former caries experience need to be considered at higher risk of recurrent caries decay. Subjects without caries experience, therefore, represent a group of special interest whose oral biofilm needs to be further characterized. Thus, the aim of the current study was to compare the composition of the oral microbiome of subjects who had never experienced symptoms of dental caries and successfully treated subjects with former caries experience using next generation sequencing. Despite comparing two healthy cohorts (that is subjects who never had experienced caries and those with former caries experience who are, however, free of untreated, active caries), we hypothesized that both groups exhibit distinct differences in the composition of supragingival plaque.

## Subjects and methods

### Subject population

The study was approved by the Human Ethics Committee of the local Medical Faculty (Ethikkommission der Medizinischen Fakultät Heidelberg, S-079/2014). All subjects gave written informed consent in accordance with the Declaration of Helsinki.

A total of 46 volunteers were recruited between 2013 and 2015 mainly from health professionals and staff at the University of Heidelberg. Thus, a homogenous healthy collective with comparable health-related behavior could be recruited (cf. Tables 1 and 2). However, the groups demonstrated a significantly different composition in age (p = 0.038) and an insignificantly different composition in gender (p = 1.05). Inclusion criteria were male or female volunteers with good general and oral health, aged 18–80 years. Exclusion criteria of subjects were as follows: active caries lesions, systemic or topical use of antibiotics within three months prior to sampling, pregnancy and breastfeeding, impaired motoric skills limiting an adequate oral hygiene and not providing written informed consent to participate in the study.

**Table 1.** Epidemiological and clinical data.

			Gender						
		Subjects	Female	Male	Age (years), mean (range)	DMFT, M ° SD	D(T), M ° SD	M(T), M ° SD	F(T), M ° SD
Group	Overall	*n* = 46	65%	35%	31.6 ± 10.7 (22–67)	3.6 ± 4.9 (0–21)	0.0 ± 0.0 (0–0)	0.28 ± 0.86 (0–5)	3.3 ± 4.4 (0–19)
	Naturally healthy	*n* = 19	79%	21%	28.1 ± 7.3 (22–52)	0.0 ± 0.0 (0–0)	0.0 ± 0.0 (0–0)	0.0 ± 0.0 (0–0)	0.0 ± 0.0 (0–0)
	Caries experience	*n* = 27	56%	44%	34.1 ± 12.2 (23–67)	6.18 ± 0.97 (1–21)	0.0 ± 0.0 (0–0)	0.48 ± 1.09 (0–5)	5.7 ± 4.49 (1–19)
U-Test (p)			0.105		0.038

**Table 2.** Descriptive statistics on health-related behavior.

			Cohort		
		Overall *n* = 46	Naturally healthy *n* = 19		Caries experience *n* = 27
Frequency of dental hygiene	>3x/d	2.2%	–		3.7%
	3x/d	8.7%	10.5%		7.4%
	2-3x/d	15.2%	21.1%		11.1%
	2x/d	71.7%	63.2%		77.8%
	1–2x/d	2.2%	5.3%		–
U-Test (p)			0.789		
Sugar containing snacks between meals	>3/d	23.9%	31.6%		18.5%
	3/d	4.3%	5.3%		3.7%
	2/d	10.9%	10.5%		11.1%
	1–2/d	30.4%	10.5%		44.4%
	1/d	15.2%	21.1%		11.1%
	<1/d	4.4%	10.5%		–
	0	10.9%	10.5%		11.1%
U-Test (p)			0.909		
Additional use of topical fluorides	Yes	47.8%	52.6%	44.4%	
	No	52.2%	47.4%	55.6%	
U-Test (p)			0.588		

The subjects underwent a clinical oral examination by professional dentists, documenting their dental and periodontal status. Following strict diagnostic criteria, cavitated lesions as well as white spot lesions with an opaque, chalky surface were defined as active caries lesions. White spots with an intact, smooth and glossy surface and brown spots were considered inactive caries lesions. All subjects were free of active caries lesions and of periodontal disease. Two subjects with caries experience showed inactive caries lesions. Four subjects with caries experience underwent invasive restorative treatment within the past year.

In accordance with the DMFT index (obtained according to Klein et al. [26]), the subjects were grouped into two categories:

1. Subjects without caries experience (***Naturally Healthy (NH)***; DMFT = 0, n = 19)
2. Subjects with ‘caries experience’ (***(CE)***; DMFT > 0 [F(T)> 0; D(T) = 0], n = 27), who had been successfully treated/had no need for invasive treatment (cf. Table 1). Within the ***CE*** group in terms of the DMFT-index, 14 subjects showed low caries experience (DMFT ≤ 4), three subjects showed moderate caries experience (DMFT = 5–7), and 10 subjects showed high caries experience (DMFT ≥ 8).

### Sampling

Sampling was performed at least 1 h after eating, with subjects having refrained from oral hygiene (this also included chewing gum or the use of mouthwash) for 48 h at that point. The collection site was isolated with cotton rolls and gently air dried. Sterile curettes were used for sampling of supragingival plaque from healthy enamel on the buccal surface of the first and second maxillary molars. The samples were pooled in an empty and sterile 1.5 ml microcentrifuge tube and frozen (−25°C) until further analysis.

### Isolation of bacterial DNA

The bacterial DNA was extracted using the QIAamp® DNA Mini Kit (QIAGEN GmbH, Hilden, Germany) as specified by the manufacturer with partial modifications to the chapter: ‘Isolation of genomic DNA from Gram-positive bacteria.’, ‘Appendix D’ on page 55 as previously described [27]. Modifications consisted of using 180 µL lysozyme (20 mg/mL) during the first lysis at 37°C for 30 min then add 20 µL of proteinase K and 200 µL of buffer AL and lyse only for 10 min without the step of 95°C for 15 min to avoid DNA degradation. DNA quantity and quality were analyzed using a NanoDrop 1000 spectrophotometer (Thermo Fisher Scientific Germany BV & Co KG, Braunschweig, Germany).

### Library preparation for next generation sequencing (NGS)

DNA was amplified using universal bacterial primers targeting the V4 region of the 16S rRNA gene (515F and 806R from Caporaso et al. [28]). Each primer was barcoded to assign the sequences to the samples. The PCR reactions mix contained Q5 High-Fidelity 1X Master Mix (New England BiolabsGmbH, Germany), 0.5 µM of each primer, 2 µL of DNA and sterile water for a final volume of 25 µL. The thermal reaction was as follows: a first denaturation at 94°C for 3 min, 30 amplification cycles (94°C for 45 sec, 50°C for 1 min and 72°C for 1 min 30 sec), and a final extension at 72°C for 10 min (cycler: Primus 25, Peqlab Biotechnologie GmbH, Germany or FlexCycler^2^, Analytik Jena AG, Germany). At the same time, proper negative controls were processed to control contamination using sterile water as template. Positive controls were also performed by processing DNA from a mock community (HM-782D, Bei resources) to control PCR and sequencing error rate. PCR products were evaluated by agarose gel electrophoresis (2%) for presence of amplicons and then purified by using Agencourt AMPure XP beads (Beckman Coulter, Germany) following the manufacturer’s instructions. Purified products were checked for quality and concentration using Quant-iT™ PicoGreen® dsDNA Assay Kit (ThermoFisher scientific GmbH, Dreieich, Germany) and Bioanalyzer (Agilent Technologies Inc., Böblingen, Germany). An equimolar mix of all the PCR products was then sent to GATC Biotech (Konstanz, Germany) which performed the ligation of the sequencing adapters to the library and the paired-end sequencing on an Illumina Miseq sequencing system with 250 cycles.

### Analysis of sequences

Paired sequences were cleaned and assembled with the R package dada2 [29]. Raw sequences were filtered and trimmed with the following parameters: maximum ambiguity: 0, number of expected errors for each read: 1, truncate reads at the first instance of a quality score less than 2. Reads were then merged as contigs and checked for chimera with the default parameters. Ribosomal sequence variants (RSV) were then assigned to taxonomy using the Silva database (Version 132). RSV assigned to eukaryotes, archae and chloroplasts were removed from the analysis.

### Statistical analyses

Descriptive indices as alpha-diversity (Shannon-Index), richness (numbers of RSVs observed), evenness (Pielou-Index) and dominance (Bergerparker-Index) were calculated using the package microbiome. Beta-diversity was assessed by calculating distance matrices based on Morisita-Horn distances. A PERMANOVA was performed to assess the statistical significance of differences between the two groups of samples. An LDA effect size (LEfSe) analysis was also performed to detect differentially abundant RSVs between groups. Correlations between the microbiota indexes, RSVs relative abundances and quantitative clinical parameters were calculated using the Spearman correlation test, and the p-values were adjusted using the Benjamini-Hochberg correction method. All statistical analyses were performed with R 3.1.4 [30].

## Results

In total 1,677,311 cleaned reads were obtained from 46 supragingival dental plaque samples and a mock community, with an average of 34,938 sequences per sample (min-max: 2,227 to 119,995). A total of 997 ribosomal sequence variants (RSVs) were found. The mean numbers of RSVs per dental plaque sample were 139 (min-max: 40–305). For the mock community, only the 22 sequence variants expected were retrieved indicating no contamination from the PCR and sequencing.

**Figure 1.**
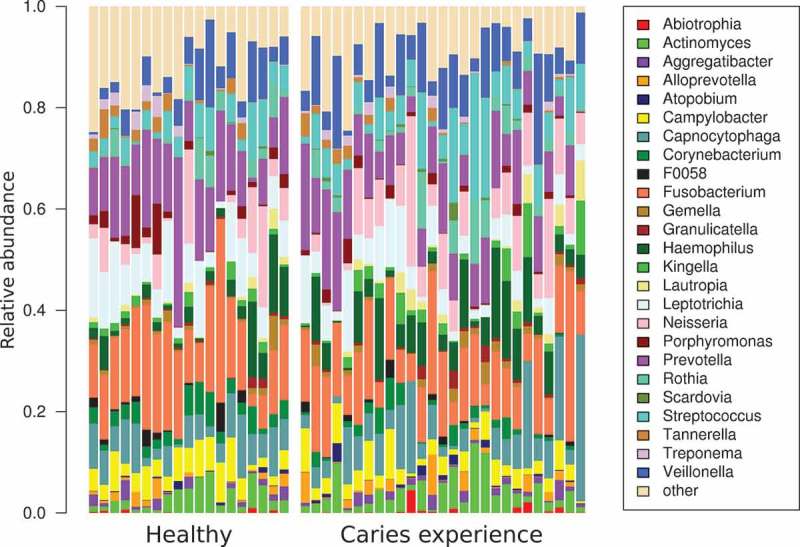
Microbiome structure at the genus level of the 46 supragingival dental plaque samples. Only the relative abundance of the 25 most abundant genera is plotted, the others are concatenated in the group named ‘other’.

The two cohorts (‘Naturally Healthy’ (***NH***) and ‘Caries Experience’ (***CE***)) exhibited a similar microbiota structure at the genus level (PERMANOVA, p-value = 0.067) (Figure 1). The most abundant genera were *Fusobacterium, Prevotella, Veillonella, Leptotrichia, Capnocytophaga, Neisseria, Streptococcus*, and *Haemophilus*; in average each of those genera accounted for more than 5% of the microbiota. However, at the RSVs level, the number of shared RSVs between the two cohorts was limited to 489 (49% of the RSVs), and only one RSV was present in all the samples (RSV11: *Veillonella* sp.). Two hundred and thirty-eight RSVs were only found in the ***NH*** cohort and 240 RSVs were found only in patients with caries experience. However, none of those RSVs were present in all the patients of the cohort. This indicated the absence of a compositional signature specific to each cohort.

**Figure 2.**
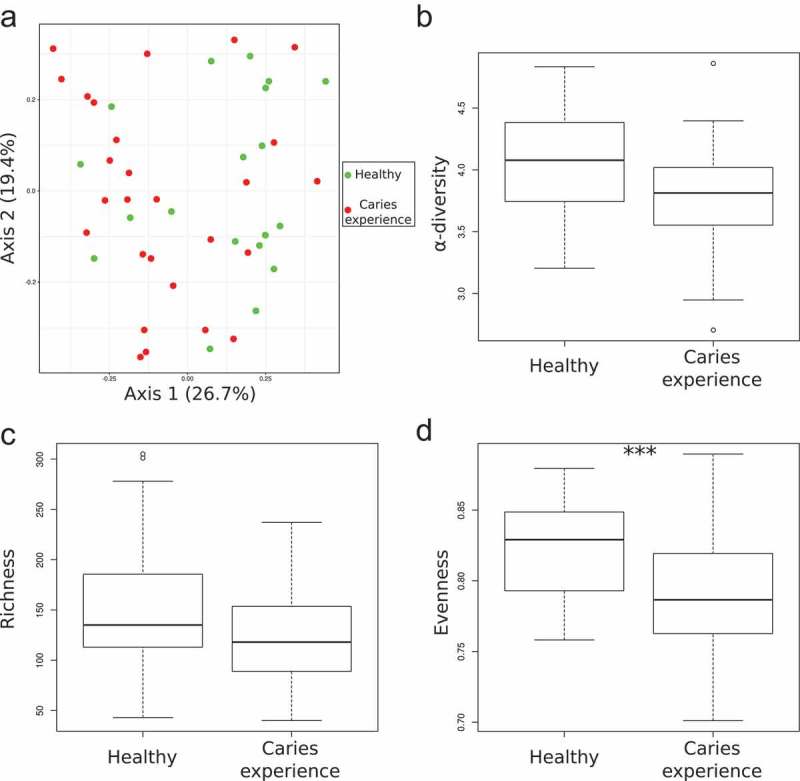
Microbiome structure of the 46 supragingival dental plaque samples differs slightly between the two cohorts. **A**) PCoA based on Morisita Horn distances, **B**) α-diversity based on the Shannon index, **C**) richness calculated as the number of observed RSVs, and **D**) evenness based on the Pielou index. Statistical differences between the two groups were estimated with a Mann-Whitney-U-Test. p-value: *** <0.001.

To analyze changes in the structure of the microbiome between the two cohorts, a PCoA based on Morisita-Horn distances was performed. The PCoA showed a slight dichotomy between the two cohorts and this observation was validated by the PERMANOVA (***NH*** vs. ***CE***: R^2^ = 0.04, p-value = 0.039) (Figure 2(a)). We observed a tendency for a lower α-diversity (Figure 2(b)) and lower richness (Figure 2(c)) in patients with caries experience. Subjects without caries experience showed a significantly higher evenness of the microbiota compared to patients with previous caries (Figure 2(d)).

The decrease of richness and evenness was linked to the difference in abundance of 12 RSVs (Table 3). One RSV belonging to the genus *Haemophilus* was increased in patients with caries experience while the 11 other RSVs were decreased significantly in this cohort. The majority of those RSVs (9/11) belonged to the abundant genera: *Corynebacterium, Fusobacterium, Capnocytophaga, Porphyromonas, Prevotella*, and *Leptotrichia*.

**Table 3.** Differentially abundant RSVs between subjects with **caries experience (CE)** and subjects without caries experience **(Naturally Healthy (NH))** by a linear discriminant analysis (LDA) effect size (LEfSe) analysis.

RSV	Ordering	LDA	*p*-Value	Taxonomy
rsv2	CE > NH	4.18	1.97E-02	*Proteobacteria; Gammaproteobacteria; Pasteurellales; Pasteurellaceae; Haemophilus*
rsv13	NH > CE	3.79	9.98E-04	*Actinobacteria; Actinobacteria; Corynebacteriales; Corynebacteriaceae; Corynebacterium matruchotii*
rsv46	NH > CE	3.66	4.39E-02	*Fusobacteria; Fusobacteriia; Fusobacteriales; Leptotrichiaceae; Leptotrichia*
rsv98	NH > CE	3.33	4.49E-05	*Bacteroidetes; Bacteroidia; Flavobacteriales; Flavobacteriaceae; Capnocytophaga ochracea*
rsv63	NH > CE	3.32	1.13E-02	*Bacteroidetes; Bacteroidia; Bacteroidales; Porphyromonadaceae; Porphyromonas pasteri*
rsv201	NH > CE	3.16	6.67E-03	*Firmicutes; Negativicutes; Selenomonadales; Veillonellaceae; Selenomonas noxia*
rsv64	NH > CE	3.13	1.37E-02	*Bacteroidetes; Bacteroidia; Bacteroidales; Prevotellaceae; Prevotella*
rsv126	NH > CE	3.10	3.35E-02	*Fusobacteria; Fusobacteriia; Fusobacteriales; Fusobacteriaceae; Fusobacterium*
rsv147	NH > CE	3.10	3.13E-02	*Fusobacteria; Fusobacteriia; Fusobacteriales; Fusobacteriaceae; Fusobacterium*
rsv97	NH > CE	3.07	3.91E-02	*Bacteroidetes; Bacteroidia; Bacteroidales; Prevotellaceae; Prevotella*
rsv287	NH > CE	3.02	2.23E-02	*Patescibacteria; Saccharimonadia; Saccharimonadales; Saccharimonadaceae*
rsv69	NH > CE	3.02	7.85E-03	*Fusobacteria; Fusobacteriia; Fusobacteriales; Leptotrichiaceae; Leptotrichia*

## Discussion

Research on the human microbiome is being coined by an overwhelming amount of data via high-throughput sequencing technologies in the past years – some authors even argue that the accumulation of data exceeds our knowledge of how to interpret them [31]. Current microbiome research often emphasizes diversity indices as the central aspect in analysis and interpretation, following the basic assumption of considering a diverse microbiome as a stable and healthy condition [31]. This hypothesis also seems to apply to caries research: Microbial diversity of dental plaque in the state of health tends to exceed that of plaque in the state of caries, with the diversity decreasing with the severity of caries [32]. Still, high diversity per se is not necessarily associated with a stable microbiome, as notified in other research areas [31,33]. Therefore, not only diversity alone but also stability is considered to be crucially important for a state of health [33]. With regard to the stability of the microbiome, the formation and development of plaque should be considered. Here, a community of early colonizers represented by *Streptococcus* spp., *Actinomyces*, and *Veillonella* spp. are considered to be predominant [34–37]. Yet, recent structural analyses indicate a prominent participation of *Haemophilus* and *Rothia* in early plaque biofilm formation [38]. In the past, *Fusobacterium* was assigned a central, bridging role by physically linking early and late colonizers [39–41]. *Fusobacterium* was considered to be a key player for a transition to disease by creating the conditions necessary for colonization of plaque through pathogens [37,42,43].

From a clinical perspective, it often remains a mystery for the general practitioner how certain subjects without any caries experience exhibit clinically sound and stable conditions in the long term and seem to be less prone to caries than other subjects exposed to comparable environmental and behavioral factors. From a cariological view, this highly interesting population must be regarded as a ‘gold-standard’ for specifying the state of health. In this context, there could not be a clearer definition of dental health than by referring to adults without any caries experience. Therefore, in our study, we compared subjects with and without caries experience. As the subjects refrained from oral hygiene for 48 h before sample collection, this represents the period of biofilm composition beyond the early stages in colonization, however still prior to highly mature biofilms being associated with disease. Thus, we sampled biofilm representing a similar state to ordinary daily plaque accumulation and that is, moreover, comparable to most other cultivation-independent analyses sampling plaque from healthy subjects (cf. 44). As our findings showed no significant difference in alpha-diversity between the two groups, these findings are in line with the hypothesis of diversity being associated with oral health and might indicate a successfully restored state of health after dental treatment in the group with caries experience. Yet, both groups exhibited a significant difference in evenness.

We could show that subjects without caries experience demonstrated significantly more *Corynebacterium matruchotii, Fusobacterium, Capnocytophaga ochracea, Porphyromonas pasteri, Prevotella*, and *Leptotrichia* (Table 3), whereas subjects with former caries experience showed *Haemophilus* being significantly more frequent (Table 3). However, recent structural analyses attribute *Corynebacterium* to be the cornerstone in supragingival plaque development [44,45]. Based on bioinformatic analyses of sequencing data from the Human Microbiome Project (HMP) and the Human Oral Microbiome Database (HOMD) Mark Welch et al. [44] identified *Corynebacterium* to have a high abundance and prevalence and to be remarkably specific to dental plaque – therefore, *Corynebacterium* was characterized as the genus most characteristic of plaque [44]. Using Combinatorial Labeling and Spectral Imaging FISH (CLASI-FISH) Mark Welch et al. [44] discovered *Corynebacterium* to be the foundational taxon of a specific multigenus consortium, which is considered to play a central role in plaque development. Its radially extending long filaments serve as anchor sites for other microbes. *Corynebacterium* is described as a base of community structure and interactions in plaque.

*Corynebacterium* is absent in the early phase of plaque development and seems to bind on preexisting biofilm being formed of early colonizers such as *Streptococcus* and *Actinomyces*. It is assumed that colonization with *Corynebacterium* takes place approximately after a 24-h stage of plaque development [44,46]. The high relative abundance and distinct specificity of *Corynebacterium* in dental plaque are thought to be attributable to its adaption strategy to dental hard tissues so that it is effectively embedded in the biofilm matrix that adheres to the tooth. From *Corynebacterium’*s ‘cemented base’ a filamentous growth outward the tooth surface contributes to the organization of the consortium and creates a protected reservoir. From this, the structure can regrow after mechanical removal by abrasion or oral hygiene [44]. Mark Welch et al. [44] discovered a highly structured, multigenus consortium built on a framework of *Corynebacterium* – Three distinct zones in the so-called hedgehog structure go along with characteristic sets of different taxa: 1) key participants of the aerobic environment of the ***perimeter*** are *Streptococcus, Haemophilus*/*Aggregatibacter*, and *Porphyromonas*; 2) the most specific and abundant in the filament-rich ***annulus*** are *Fusobacterium, Leptotrichia*, and *Capnocytophaga*; 3) the **base** is dominated by *Corynebacterium*.

Along with *Corynebacterium* we found *Fusobacterium* to be more abundant in the ***NH*** group. In general, *Fusobacterium* is more abundant in subgingival than in supragingival plaque and is regarded as being anaerob. Nevertheless, microaerophile supragingival-abundant subspecies have been identified to grow efficiently in low-oxygen environment as well [42,44,47–51]. In relation to the aforementioned model of the hedgehog structure, the oxygen-poor and CO_2_-rich environment of the annulus suggests suitable conditions for the proliferation of microaerophilic strains of *Fusobacterium*. In this context, the relatively high prevalence of *Fusobacterium* in the ***NH*** group might be a consequence of the high abundance of *Corynebacterium* providing a suitable habitat. Similarly, the relatively high abundance of *Capnocytophaga* and *Porphyromonas* in the ***NH*** group might be connected to the framework given by the hedgehog structure.

Due to its central role in plaque development, some authors propose targeting *Corynebacterium* specifically for novel antimicrobial therapies [1,45,52]. According to these authors, a new antiplaque approach could be based on inhibiting the growth of *Corynebacterium* to prevent settlement of late-colonizing pathogens and thus to inhibit the formation of a mature biofilm community [53].

Contradictorily to this approach, our findings allowed a contrary interpretation: With *Corynebacterium* being overrepresented in subjects without caries experience, we are in line with other studies in which *Corynebacterium* was also identified as being health-related [32,54]. Gross et al. [54] compared the bacterial community composition of supragingival plaque in the state of health and severe caries in young permanent dentitions. *C. matruchotii* was found to be significantly decreased in subjects with active carious lesions (n = 21) compared to the naturally healthy control subjects without caries experience (n = 18). Xiao et al. [32] analyzed supragingival plaque samples from naturally healthy subjects without caries experience (n = 29) and subjects with dental caries (n = 131). According to the DMFT-Index, the subjects were grouped into four categories: naturally healthy subjects without caries experience/‘No-caries’ (DMFT = 0, n = 21), ‘Low-caries’ (DMFT ≤ 4, n = 32), ‘Moderate-caries’ (DMFT = 5–7, n = 37), and ‘High-caries’ (DMFT ≥ 8, n = 62). Here, *Corynebacterium* was also found to be significantly more abundant in subjects without caries experience. In this context, the authors tended to consider *Corynebacterium* as a possible signature species for dental health [32,54] and stability. Solely reducing health to the abundance of a single species has to be considered incommensurate with the complex multifactorial nature of caries, as it showed to be insufficient to relate the state of health and disease on the mere absence or presence of *S. mutans*. Nevertheless, the strong correlation between the abundance of *Corynebacterium* and the ***NH*** group – exhibiting stable and sound conditions and a very low caries risk – was unexpected. Although the majority of subjects in the ***CE*** group showed only a relatively low caries experience with DMFT-indices <4, we were still able to find a relation with the ***NH*** group that confirmed previous findings linking *Corynebacterium* to dental health. Unlike previous studies, we compared two healthy groups, both composed of subjects without active caries. Other studies comparing the oral microbiome with caries experience either did not include any naturally healthy controls that never had any caries symptoms [55,56], or compared them to subjects with active carious lesions [32,54,57,58]. To the best of our knowledge, this is the first attempt to compare supragingival plaque composition between caries-free subjects without any history of caries and caries-free individuals, who had undergone invasive restorative treatment in the past. Nevertheless, certain limitations of this pilot study must be acknowledged. Since this study was exploratory and due to our strict inclusion criteria, we could include only a relatively small number of subjects. This is accompanied by an unequal distribution between number of subjects and an unequal age and gender distribution between the two groups. While representative epidemiological data in Germany show no significant differences in the gender distribution in naturally healthy adults without any caries experience [59], the ***NH*** group in our study exhibited a female to male ratio of 15:4. Finally, it should be noted that a single analysis of the composition of dental plaque lacks important information on the possible influence of *Corynebacterium* on diversity and stability, in particular with regard to its unique structural features.

In line with our results, Xiao et al. [32] found *Corynebacterium* in addition to *Fusobacterium* and *Leptotrichia* as health-related genera significantly enriched in subjects without caries experience. Missing comparable analyses between healthy subjects with and without caries experience, in the ***CE*** group the high abundance of *Haemophilus* – being predominant bacteria in early plaque biofilm formation – appeared remarkable, yet ambiguous to interpret. Following the same protocol for sample collection with a refrained oral hygiene for 48 h, both groups should exhibit a biofilm beyond the early stages. Consequently, the following hypothetical question arises: Might an over-representation of *Corynebacterium* promote a diverse and stable microbiome and thus contribute to resilience in the healthy microbiome? A speculative attempt to give an explanation may be found in relation to *Corynebacterium’*s structural characteristics – with *Corynebacterium’*s attachment sites on the tooth surface being protected from abrasion or oral hygiene. Rapidly regrowing filaments might mediate the formation of a stable, healthy microbiome. Obviously, these questions are based on deductive and mechanistic assumptions and cannot be answered with our study design and methods used; still, our results stand out from others and can be contextualized with recent findings on the formation and structure of dental plaque – representing an intriguing point of discussion.

For a comprehensive and thorough understanding of variations in microbiome composition and diversity, as well as possible associations with states of health and disease, future efforts in caries research should be directed to an integration of genomic methodologies and structural analysis [1,31]: There is an urgent need for well-designed longitudinal clinical studies investigating community structure, composition and stability, as well as the association between the composition and the function of oral microbiota. Further efforts to evaluate the possible central role of *Corynebacterium* in plaque formation will need to comprise multiple disciplines in a holistic approach beyond mere microbiome composition. This would allow further insight into its value as a risk or protective factor or therapeutic target.

## Data Availability

Sequence data, metadata files and R script used for analysis have been uploaded and are publicly available in Figshare (https://figshare.com/s/2eb93396f96f539bdf0c).
